# Human-Robot Perception in Industrial Environments: A Survey

**DOI:** 10.3390/s21051571

**Published:** 2021-02-24

**Authors:** Andrea Bonci, Pangcheng David Cen Cheng, Marina Indri, Giacomo Nabissi, Fiorella Sibona

**Affiliations:** 1 Dipartimento di Ingegneria dell’Informazione (DII), Università Politecnica delle Marche, 60131 Ancona, Italy; g.nabissi@univpm.it; 2 Dipartimento di Elettronica e Telecomunicazioni (DET), Politecnico di Torino, 10129 Torino, Italy; pangcheng.cencheng@polito.it (P.D.C.C.); marina.indri@polito.it (M.I.); fiorella.sibona@polito.it (F.S.)

**Keywords:** human-robot perception, human-robot collaboration, collision detection, human action recognition, collision avoidance, machine vision, 3D sensors, robot guidance

## Abstract

Perception capability assumes significant importance for human–robot interaction. The forthcoming industrial environments will require a high level of automation to be flexible and adaptive enough to comply with the increasingly faster and low-cost market demands. Autonomous and collaborative robots able to adapt to varying and dynamic conditions of the environment, including the presence of human beings, will have an ever-greater role in this context. However, if the robot is not aware of the human position and intention, a shared workspace between robots and humans may decrease productivity and lead to human safety issues. This paper presents a survey on sensory equipment useful for human detection and action recognition in industrial environments. An overview of different sensors and perception techniques is presented. Various types of robotic systems commonly used in industry, such as fixed-base manipulators, collaborative robots, mobile robots and mobile manipulators, are considered, analyzing the most useful sensors and methods to perceive and react to the presence of human operators in industrial cooperative and collaborative applications. The paper also introduces two proofs of concept, developed by the authors for future collaborative robotic applications that benefit from enhanced capabilities of human perception and interaction. The first one concerns fixed-base collaborative robots, and proposes a solution for human safety in tasks requiring human collision avoidance or moving obstacles detection. The second one proposes a collaborative behavior implementable upon autonomous mobile robots, pursuing assigned tasks within an industrial space shared with human operators.

## 1. Introduction

The perception capabilities of robots will gain ever-greater importance in the next smart factories. The robot has been gaining an increasingly important role within factories and warehouses for decades, recently witnessing a boost in its use as a support to human workers, as a team member or a flexible part of manufacturing processes. Autonomous and collaborative robots will be increasingly involved in operations requiring a shared working space with human actors. Most of the activities will have to be done avoiding obstacles, working collaboratively with human beings, autonomously locating and identifying the parts to be worked or moved. This perspective of collaborative environment between humans and robots in production settings goes beyond the concept of Cyber Physical Production System (CPPS) [1,2]. In CPPSs, a smart production plant is itself a Cyber Physical System (CPS) integrating cyber aspects as computation, communication, control, and networking technologies into the underlying physical system. A CPS can quickly react and adapt to market changes negotiating production resources as in [3], or using some intelligent reasoning tools as suggested in [4], but humans are generally considered to be intruders into the automated tasks. CPSs should be able to reprogram their activities reacting to the presence of humans or other mobile systems, but generally they do not interact or collaborate actively with them. This leads to conceive the next future evolution of the CPSs towards Cyber Physical Human Systems (CPHSs) [5,6], where the control, communication and automation technologies, physical plants and humans must pursue a common goal. The latter fact opens up new challenges with respect to the conventional interpretation of CPSs, where humans were very often considered to be independent passive entities that operate, use or consume the CPS resources. This also motivates the research for new solutions for developing trustworthy, safe, and efficient Human-Robot (HR) perception to achieve an enhanced HR Interaction (HRI) in collaborative work environments, thus allowing the development of CPHSs. In the context of CPHS, the adoption of HR teaming is still hindered by the lack of clear guidelines for safety, interfaces and design methods [7]; the HR Perception (HRP) step and its requirements are then fundamental to successfully implement the paradigm of CPHSs as the core of the Factory of the Future (FoF). The digitalization of the whole manufacturing system requires managing plenty of heterogeneous sensors able to share and fuse the information provided by other sensors, as well as increasing capabilities not limited to detection. Indeed, a high technological level is needed by the sensors, which have not only to read the data and reduce the noise effects, but also to process them (edge computing) to enable predictive maintenance operations [8]. In such a scenario, the availability of sensors for the HRP becomes a key issue for managing the operations of HRI in the FoF. The choice of the type of perceptive system to be used is highly related to the task to be fulfilled, to the level of autonomy to be guaranteed, and to the kind of HRI that must be established. It is worth highlighting that there exist three types of HRI in the industrial scenario [9]:

- HR Coexistence, where humans and robots share the same working space, but performing tasks with different aims; here, the human is perceived as a generic obstacle to be avoided, and the robot action is limited to collision avoidance only.
- HR Cooperation, in which human and robots perform different tasks but with the same objectives that should be fulfilled simultaneously in terms of time and space. In this scenario, the collision avoidance algorithm includes human detection techniques, so the robot can differentiate the human operator from a generic object.
- HR Collaboration (HRC), where a direct interaction is established between the human operator and the robot while executing complex tasks. This can be achieved either by coordinated physical contact or by contactless actions, such as speech, intentions recognition, etc.

This paper analyzes several sensors and perception techniques adopted for HRI applications, robot guidance and collision avoidance for all the main types of robotic systems commonly used in industry, such as fixed-base manipulators, collaborative robots (cobots), mobile robots and mobile manipulators. The analysis investigates how these robotic systems perceive the presence of human operators and how they react for cooperative and collaborative applications. Various applications, strongly relying on HRP to achieve HRC, are reviewed with a particular focus on the handled type of data, the need to fuse the information that comes from different sensors to guarantee an efficient and safe HRI, as well as the specific requirements of the perception tasks (e.g., the perception range, the safety issues, the environmental influences, etc.). Particular attention is devoted to vision and distance sensors, which are the most employed for human perception in various types of robotic systems. Monocular RGB (Red Green Blue), stereo, RGB-D (Red Green Blue-Depth), and more recent event-based cameras stand out among the vision sensors, whereas the most used distance sensors are based on light scan technology, such as the LIDAR. The goal of this survey is to provide useful information to accomplish several robotic tasks in HR collaborative industrial environments. The aim is to offer to the reader a quite complete overview of the different solutions proposed in the literature and of the modalities with which they have been applied to different types of robotic systems. The carried-out analysis provides detailed and aggregated information about the various types of sensors adopted to handle the presence of human operators in several industrial scenarios, as well as about the sensors and algorithms combinations that seem to offer the best performance. The paper is completed by the introduction of two proofs of concept, developed by the authors, for possible collaborative robotic applications based on enhanced capabilities of human perception and interaction. The first proof of concept is relative to the problem of human and obstacle detection for collision avoidance in an HRC application with a fixed-base collaborative robot: thanks to a two-fold use of an RGB-D sensor, human safety can be achieved, overcoming the current limits related to sensor accuracy and efficient execution of collision avoidance algorithms. The second proof of concept proposes a collaborative extension of an architecture of autonomous mobile robots, active in an industrial space shared with human operators. In the basic version of this architecture, the agents can move guaranteeing the safety of the human operators encountered during the motion, but without any type of collaboration with them. In the envisaged extension, the agents act as collaborative mobile robots, able to recognize trained operators and perform collaborative actions, directly requested by the operators through a pre-defined sequence of movements, properly interpreted by the robots. The remainder of the paper is organized as follows: Section 2 investigates how the main types of robotic systems perceive the human presence and how this is handled using different sensors; a brief overview of human and environment perception in the industrial context is also provided. Section 3 illustrates the two proofs of concept, developed to investigate possible future improvements for collaborative robotic applications, thanks to enhanced capabilities of human perception and interaction. Section 4 finally draws conclusions and sketches future trends.

## 2. Robotic Systems and Human-Robot Perception

In this section, several types of robotic systems commonly used in industry (i.e., fixed-base manipulators, cobots, mobile robots and mobile manipulators) are considered with the aim of illustrating how they perceive and react to the presence of human operators or static obstacles in the industrial scenarios, for cooperative and collaborative applications. The section is completed by a summarizing overview of the most adopted types of sensors and HRP methods.

### 2.1. Fixed-Base Manipulators and Cobots

HRC in the context of fixed-base robots is a topic of great interest in recent research. In the various applications in which humans and robots coexist in the same environment, two scenarios may be considered:

- A full awareness of the human presence and the environment is necessary.
- Only the safe management of the shared spaces can be sufficient, guaranteeing that humans cannot be injured during the robot motion.

In the first case, different types of sensors are used to track humans or obstacles in the manipulator workspace, obtaining a complete 3D model of the environment. In such a way, it is possible to monitor the distance between the robot and any object in the workspace, whereas a high-level controller can re-plan the robot trajectory to avoid collisions or stop the system, if necessary. In the second case, collisions are generally detected estimating the dynamic properties of the robot, together with the information coming from the proprioceptive sensors, which industrial robots are usually equipped with. The robot motion is then re-planned and controlled to limit the contact forces, so that possible collisions with humans or obstacles are no more critical.

This section investigates the enhancements achieved in recent HR applications mainly using exteroceptive sensors, without leaving out the available proprioceptive-based methods to estimate the robot contact forces through the knowledge of the robot dynamics. Without the use of exteroceptive sensors, the robot has not a perception of the 3D external environment: the interaction contact forces are kept limited, leaving the robot unaware of the presence of humans and obstacles. Therefore, by using proprioceptive sensors only, collisions are not avoided, but they become not dangerous for humans. Currently, commercial cobots mainly adopt methodologies that limit forces, avoiding the use of vision sensors. Thanks to their smooth surfaces and operating velocities that are adequate to the collaboration with humans, cobots work *together with* the operator, even if they actually do not perceive his/her presence, but only the possible contact. Some solutions have been recently proposed to also make the traditional manipulators able to establish some kind of collaboration with humans, according to ISO/TS 15066:2016 [10,11]. The method proposed in [12] limits the force for a traditional industrial manipulator, and detects collisions without the use of external sensors. It adopts time-invariant dynamic models and supervised feed-forward input-delay neural networks on signal processing to estimate the required current signals for a given robot motion. The predicted current signals are then compared with the actual absorbed motor currents, which are continuously measured by the robot controller; a collision is then detected when the current required by the manipulator is greater than the predicted one. Another approach is proposed in [13], which avoids the use of external force sensors, generally not present in standard manipulators, and exploits the dynamic model of the robot in both dynamic and quasi-static modes to detect the external forces.

To overcome the current limits in HR applications, the use of exteroceptive sensors, in particular vision sensors, can be a valid solution, even if there are still problems related to the accuracy and repetitiveness. Indeed, vision sensor performance is highly affected by environmental conditions, such as exposure, brightness, reflectiveness, etc. However, such sensors are the most suitable for providing the robot complete awareness of the environment, to avoid obstacles and re-plan trajectories. For this reason, vision sensors are generally used to check the workspace, allow humans safety and detect the presence of objects, despite their critical issues.

The most used vision systems for HR collaborative applications are stereo cameras, RGB-D (Red Green Blue—Depth) cameras, proximity sensors or laser scanners. Obstacle tracking data can be used to estimate human intentions, to create models of the 3D environment, to calculate distances between the robot and the obstacle, to integrate data coming from virtual and real world to test an application in simulation, and so on. This section reviews the current state of the art with a particular focus on the three fundamental aspects for the development of HR applications:

- Type of sensor: the sensor output depends on the sensor technology. Therefore, the algorithms used to process the data can be quite different.
- Methodology to detect obstacles in the scene: the chosen methodology depends on the type of sensor but also on its location. The sensor can be positioned somewhere in the environment to monitor the entire scene or can be mounted on the robot arm. In the first case, it is necessary to distinguish humans and obstacles from the manipulator, otherwise the robotic system can identify itself as an obstacle. In the second case, the sensor position is not fixed and must be estimated to recreate the 3D scene.
- Anti-collision policy: once the obstacle is detected on the robot path, and hence the risk of a possible collision, the robot can be stopped providing some warning (e.g., sounding an alarm) or its trajectory can be automatically re-planned to avoid the obstacle.

Several works propose the use of a Kinect RGB-D sensor, which provides RGB and depth space images to reconstruct the 3D environment. In [14], the Kinect sensor is used to generate 3D point cloud data and to study the collision prediction of a dual-arm robot (Baxter). To detect obstacles in the scene and prevent self-collision avoidance, the authors proposed a self-identification method based on the over-segmentation approach using the forward kinematic model of the robot. To improve the processing speed, a region of interest is determined based on the skeleton of the robot, then a collision prediction algorithm estimates the collision parameters in real time for trajectory re-planning. Flacco et al. [15] presented a fast method to calculate the distance between several points and moving obstacles (e.g., between robot joints and a human) in depth space with multiple depth cameras (Kinect). The robot kinematics is used to identify the point cloud data representing the robot itself to eliminate it from the scene. The distance is used to generate repulsive vectors that control the robot while executing a motion task, thus achieving a collision avoidance application. Also, in [16], the Kinect sensor was used to add data coming from real obstacles in a virtual scene, where the robot is modelled. This approach aims at testing re-planning algorithms and HR interaction in safe conditions, simulating possible scenarios where humans and robots must collaborate. However, in all these works the Kinect sensor shows its limits in terms of accuracy and reliability. In [17], a method was proposed to improve the accuracy of the Kinect sensor merging real and virtual world information; in particular, some accuracy problems are overcome using a skeletal tracking approach. A highly detailed avatar is created to represent human behavior in the 3D scene, consisting of thousands of polygons. Then, the Kinect sensor is used as an input device for skeletal tracking and positioning of the user. Nevertheless, there are different types of low-cost RGB-D cameras; useful information regarding the choice among the most used in research can be found in [18], where sensor performance is compared in an agriculture application.

The use of a simple RGB camera to detect obstacles was proposed in [19], in a case study in which an industrial manipulator is used. The robotic system is provided with smart sensing capabilities, such as vision and adaptive reasoning, for real-time collision avoidance and online path planning in dynamically changing environments. The machine vision module, composed of low-cost RGB cameras, uses a color detection approach based on the hue saturation value space to make the robot aware of environmental changes. This approach allows the detection and localization of a randomly moving obstacle; the path correction to avoid collision is then determined by exploiting an adaptive path planning module along with a dedicated robot control module. It must be underlined that using only a standard RGB camera, the obstacles detection can be performed in 2D assuming a constant height along the third direction. This solution may be valid for manipulators employed for simple pick-and-place tasks and it can be executed in a fast-working cycle.

A different solution, which integrates sensors used for virtual world interaction, is proposed in the field of robotics surgery, where any possible collision between the robot and the medical staff is considered to be critical [20], but some of its characteristics could be exploited in different contexts, such as the manufacturing one, for applications requiring a strict HR collaboration. The HTC VIVE PRO controllers are used as an Internet of Things technology to measure the distance between surgeons and the robot. When the distances between humans and the robot, measured through the smart controllers, become critical, a virtual force is applied to the manipulator to move the robot elbow in a spare workspace. This avoids the direct hands-on contact of the surgical robot arm by applying the virtual force to move the swivel angle of the KUKA iiwa. Due to the kinematic redundancy of the manipulator, a swivel motion with the robot elbow can be performed without moving the robot tool pose avoiding compromising the surgical intervention. In [21], the same authors previously investigated the cartesian compliance strategy that involves online trajectory planning to avoid violation of some defined constraints.

A novel sensor proposed in [22], which consists of skins with proximity sensors mounted on the robot outer shell, provides an interesting solution to occlusion-free and low-latency perception. The collision avoidance algorithms, which make extensive use of these properties for fast-reacting motions, have not yet been fully investigated in this work. A collision avoidance algorithm for proximity sensing skins is proposed as a first solution by formulating a quadratic optimization problem. The authors point out that compared with common repulsive force methods, the algorithm confines the approach velocity to obstacles and keeps motions pointing away from obstacles unrestricted.

It is worth noting that a good HRC requires good HR interfaces and the possibility for the human operator to easily establish some kind of communication with the collaborative robot [23]. A proper use of adequate sensors is fundamental to this aim. Cameras can be employed, but better results can be achieved integrating also specific sensors such as the Leap Motion, which can be used to recognize coded gestures of the operator as input commands to the robotic systems (e.g., as for the teleoperated robotic arm in [24]), but also to enhance the perception capabilities provided by cameras, as in [25]. Here, a multi-source heterogeneous vision perception framework is proposed to acquire information about the human workers in various conditions and on the working environment during HRC tasks in manufacturing. The proposed system includes RGB-D cameras (i.e., Kinect sensors), located around the working area to produce 3D point cloud data, and Leap Motion sensors on the workbench to track the worker’s hands. In this way, a wide and clear perception is achieved of both the working area and the worker.

In [26], a system composed by five Inertial Measurement Unit (IMU) sensors is used to recognize human gestures. The IMU sensors are distributed in the upper part of the operator’s body, along with an ultra-wide band positioning system. The latter activates the collaborative mode when the human operator is in close proximity to the robot. Static and dynamic gestures used to command the robot are processed and classified by an Artificial Neural Network (ANN). A similar work related to gestures in the industrial context is presented in [27], in which IMUs and a stereophotogrammetric system are used to track and analyze the human upper body motions, in particular when he/she picks and places several objects at different heights. The gestures sequences are collected in a database, and can be used to optimize the robot trajectories and guarantee the safety of the human operator.

A sensor data fusion algorithm is proposed in [28] to estimate and predict the human operator occupancy within the robot workspace. The algorithm merges the information coming from two different depth sensors, a Microsoft Kinect and an ASUS Xtion, defining a set of swept volumes that represents the space occupied by the human. In this way, the motion of the robot can be re-planned to be compliant with the safety constraints, thus avoiding any collision with the human operator.

More insights into hand gestures recognition by means of the Leap Motion and other solutions for HR interaction are provided in the following subsections.

### 2.2. Mobile Robots

**Figure 1:**
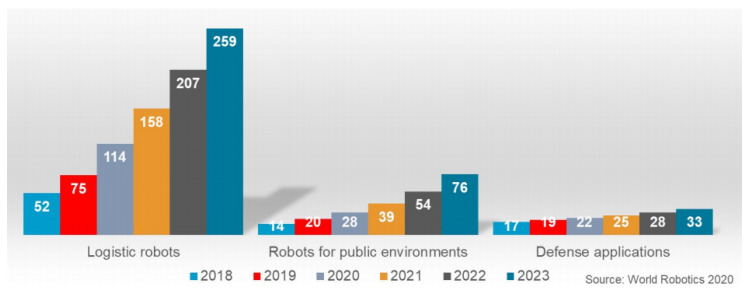
Service robots for professional use. Top 3 applications unit sales 2018 and 2019, potential development 2020–2023 (thousands of units) [30].

Mobile robotics is gaining ever increasing importance within the industrial context. Indeed, Industrial Mobile Robots (IMRs) represent essential elements of the present and future production line and logistics workspaces (Figure 1). Specifically, Autonomous Mobile Robots (AMRs) allow the improvement of flexibility of the working setup, since they get rid of the path constraints of classical Automated Guided Vehicles (AGVs). Spatial and temporal flexibility, when considering production plants, can improve productivity and reduce overall downtime, for example, when the production sequence configuration must be changed. Indeed, flexibility requirements (dictated by recent market demands for custom products) inevitably affect the current and future production line design [29].

It is clear that the optimization of processes has a pivotal role for the overall efficiency (e.g., productivity, energy consumption) of a working setup, as discussed in Section 1 with reference to the well-established CPS concept. IMR perception of its surroundings thus acquires relevance for achieving optimal integration with other CPS elements, going beyond the basic role in the localization of the platform during navigation. In particular, autonomous navigation of AMRs introduced a further need for effective HRP approaches. Indeed, for what concerns cooperative operations, effectiveness may be undermined by the perception that human operators have of moving autonomous agents. Conversely, the mobile base task execution could be slowed down by ill-managed perception of humans. As a matter of fact, the pre-definition of AGV motion paths guarantees predictability in opposition to the AMRs motions, which are often hard for a human operator to interpret. The perception systems of traditional AGVs [31] have undergone heavy changes [32,33] to achieve navigation autonomy and advanced perception of the environment and humans in industrial scenarios [34], favoring the investigation of real-time approaches [35]. Moreover, due to the gradual and now extensive use of fixed-base collaborative robots along the production line, the implementation of safe collaborative operations using IMRs has been attracting a lot of interest. Industrial mobile platforms then need to elevate their perception level from a merely informative approach to a semantic interpretation of the robot surroundings.

Despite advanced perception of humans seems to be an emerging topic within the industrial context, it has already been widely explored and adopted in other fields, from assistive service robotics to agricultural ones, where robotics plays a significant role in the process chain. The authors consider relevant and interesting for this review to report some of the approaches to HRP developed in these fields, since it is not unlikely that the FoF will implement similar or comparable approaches on intelligent IMRs. In [36], a non-intrusive solution to robot aware navigation is presented, which lets the user preferences determine the robot behavior in a domestic workspace sectioned in virtual areas. In [37], the human and the mobile robot share a common task, since the robot is teleoperated by an operator, whose visible 360-degree scene is enriched by interactive elements drawing the attention to information-rich areas; a 360-degree camera is exploited, and its frames are processed using the You Only Look Once (YOLO) Convolutional Neural Network (CNN)-based framework [38]. In this case, the goal achievement is common, and the perception of the human operator and the robot somehow enhance each other. Also, in [39] teleoperation is implemented, using a hybrid shared control scheme for HRC. The operator sends commands to a remote mobile robot using an electromyography (EMG) signal sensor to reflect muscle activation; the human partner is provided with a haptic device, which receives a force feedback to inform about the existence of an obstacle. The work presented in [40] aims at highlighting challenging natural interactions between a mobile robot and a group of human participants sharing a workspace in a controlled laboratory environment, demonstrating that humans follow less jerky and irregular paths when navigating around one autonomous navigation condition than around a teleoperated robot. The experiments are performed on autonomous mobile robots using optimal reciprocal collision avoidance, social momentum and teleoperation as navigation strategies. In [41], an approach named RObot Perceptual Adaptation (ROPA) is proposed. This algorithm learns a dynamical fusion of multi-sensory perception data, capable of adapting to continuous short-term and long-term environment changes; a special focus is set on human detection, based upon different types of features extracted from color and depth sensors placed on the mobile robot, with the aim of achieving long-term human teammate following. A structured light camera is used for color-depth data and a digital luminosity sensor for luminosity data. Similarly, in [42], the authors introduced a representation learning approach that learns a scalable long-term representation model, for scene matching. The features of multiple scene templates are learned and used to select, in an adaptable way, the most characteristic subset of templates to build the representation model for the current surrounding environment. The latter procedure is performed with the aim of implementing long-term delivery of information in collaborative HRP applications, taking advantage of Augmented Reality (AR). Furthermore, what seems clear from works reviewed within the agricultural field, concerning collaborative applications and relative perception between humans and robots, is the focus on safety without leaving out comfort of the interaction [43,44]. The work presented in [45] proposes a planning model based on RNNs (Recurrent Neural Networks) and image quality assessment, to improve mobile robot motion in the context of crowds. Acquired images are pre-processed exploiting OpenCV (Open Computer Vision) calibration tools and then the background noise is filtered out using the designed RNN-based visual quality evaluation. Additionally, concerning the assistance service robotics context, the bidirectional meaning of perception is particularly evident, since the robot should be perceived by users as naturally as possible, and the robot itself must have capabilities of intention recognition to be actually of some utility to the human counterpart, e.g., in Sit-To-Stand assistance [46]. Moreover, the SMOOTH robot project, presented in [47], provides an example of adaptive sensory fusion computed via a single multi-sensory neuron model with learning, to boost perception of human capabilities of a welfare robot. The robot is equipped with a front safety laser scanner and two cameras, one front and one back facing. Finally, the survey presented in [48] highlights the importance of data fusion to enhance the perception capability of mobile robots. The reviewed works consider data coming from multiple sensors (e.g., LIDAR, stereo/depth and RGB monocular cameras) to obtain the best data for the tasks at hand, which in this case are autonomous navigation tasks such as mapping, obstacle detection and avoidance or localization.

Given these example approaches, it is easy to envision how they could greatly impact the emerging HRP research in the industrial context. Many algorithms are being developed with the aim of being ideally applicable in any context involving humans and robots. The need for a unified framework to enable Social-Aware Navigation (SAN) is stressed in [49], where the authors propose a novel approach for an autonomously sensed interaction context that can compute and execute human-friendly trajectories. They consider several contexts and implement an intent recognition feature at the local planning layer.

For what concerns the industrial logistics context, the authors of [50] propose a range finder-based SAN system to implement collaborative assembly lines with a special emphasis on human-to-robot comfort, considering the theory of proxemics. A cost function is assigned both to assembly stations and operators to affect the cost map for the mobile robot navigation. In [51], a human-aware navigation framework is proposed, to work within logistics warehouses. The simulated mobile robot is equipped with a laser scanner and an RGB-D camera to detect a person and estimate the pose to consider it as a special type of obstacle and avoid it accordingly. The proposed strategy is made up of 2-steps: (i) the use of the depth information for clustering and identifying 3D boxes that are likely to enclose human obstacles, then (ii) the computation of a confidence index for human presence based on the RGB data. Instead, the approaches proposed in [52] aim at demonstrating the integration of AR as an enabler for enhanced perception-based interactions along assembly Manufacturing Execution Systems (MES). The authors propose an application involving mixed reality smartglasses for AR implementation for collaboration with a cobot, and a path visualization application for humans working with AGVs, using an AR computing platform. Another work proposes a solution to HRI using (i) gesture control and eye tracking technologies for the robot to interpret human intentions, and (ii) a pocket beamer to make robot information interpretable by the human operator [53]. Finally, in [54] the authors propose an HR skill transfer system: a mobile robot is instructed to follow a trajectory previously demonstrated by a human teacher wearing a motion capturing device, an IMU in this case. A Kinect sensor is used for recording the trajectory data, used to model a nonlinear system called a Dynamic Motion Primitive. Then, exploiting multi-modal sensor fusion, the pose and velocity of the human teacher undergo a correction process and a novel nonlinear model predictive control method is proposed for motion control.

### 2.3. Mobile Manipulators

Manipulators have been employed in many applications, increasing the efficiency in the industrial production line. However, these are usually located in fixed positions along the line, which is a limitation for some applications that need to cover large working spaces, as in the automotive or aerospace industry. To overcome this problem, it is possible to rely on mobile manipulation. A manipulator attached to a mobile platform improves the flexibility for many tasks, since the redundancy offered by a mobile manipulator allows the planning of human-like motions while avoiding singularity configurations. Due to the mobility advantages, it is also used for intralogistics and service robotics applications [55,56].

Most of the mobile manipulators available on the market consist of a combination of a collaborative lightweight manipulator and a mobile platform. The mobile platform in these cases may be collaborative or not. It is worth highlighting that currently there are no safety standards specific to these hybrid systems so, in order to be compliant with collaborative operations and safe constraints with mobile manipulators, a combination of two or more standards should be considered, e.g., ISO/TS 15066 [10] and/or ISO 10218-1 [57] for manipulators, and ISO 3691-4 [58] for mobile robots.

Since the collaborative manipulator itself was designed for collaborative applications, it can react to the physical contact of the human operator with no harm. However, to allow the robot to perceive better its environment, and therefore improve the decision-making process for the motion planning needed for a specific task, other sensors may be integrated to the robot. The way the robot may sense its surroundings and the way it reacts to the human actions strongly depend on the application. In fact, there are applications in which vision sensors are widely used to emulate the decision making based on the human vision.

For example, the mobile manipulator proposed in [59] is designed for HRC tasks, in which object detection and manipulation are considered to be critical skills. According to the authors, using an RGB-D camera is more robust than using stereo vision cameras, since the latter ones only rely on image features. The images and videos coming from RGB-D cameras are also useful for either (i) configuring the motion constraints based on the human presence, differentiating human-type obstacles from the generic ones, or (ii) predicting the human activity, so the robot can react accordingly to the operator action [60]. A sensor system that provides reliable 2½D data for monitoring the working space is presented in [61]. The system elaborates data coming from three pairs of grayscale stereo vision cameras and a Time-of-Flight camera that monitors the motion of the human operator collaborating with the manipulator. The area monitored by the sensor system corresponds to the safety zone, in which specific actions of the robot are enabled when the hand of the human operator is close to the manipulator tool.

The mobile manipulator proposed in [62] uses two sensors that perceive the environment: an RFID sensor that lets the robot know where the objects are in the space and an RGB-D camera that identifies tags with unique IDs, which contain semantic information and properties of the world entities. The paper did not specify if the robot is working with a human or not, but the interesting fact is that the robot can learn from experience, and each time it must perform an action, the motion planning comes from experiential knowledge and the geometric reasoning for doing such task.

To give more information to the robot regarding the human intentions or actions, gestures and speech are commonly used for controlling a robot. Nevertheless, hand gestures are preferred over speech, since the industrial environment is often noisy, and so verbal communication is difficult [63]. The gesture recognition is performed by analyzing two features from an RGB-D camera: a convolutional representation from deep learning and a contour-based hand feature. This permits the robot to recognize the hand gestures of the human and execute specific commands. Moreover, the same authors proposed alternative methods for human tracking [64], such as applying multi-sensor integration (for example, mounting low costs laser range finders and camera systems at specific poses) and using laser readings and train the tracking system according to human body patterns. The authors in [65] suggest that a 3D sensing system is important for human detection and for understanding the behavior. In that regard, a redundant sensory system, such as a combination of 2D laser scanners and sensors that reconstruct the environment in 3D using stereo vision, may ensure safety and be compliant with the ISO 10218-1 and ISO/TS 15066 regulations, which are related to safety for collaborative robots. Nevertheless, those standards involve collaborative manipulators, so the safety concerning the mobile platform should be also considered, as discussed in [66] that analyzes the possible hazards of mobile robotic systems in industry and proposes some countermeasures for those risks. Therefore, the use of sensor fusion or artificial intelligence-based methods are suggested, since they increase the coverage of the information from different sensors and overcome safety problems.

A framework referred as ConcHRC [67], which represents an extended version of the previous FlexHRC framework [68], allows the human operator to interact with several robots simultaneously for carrying out specific tasks. The architecture is composed of three layers: perception, representation and action. In particular, the perception layer elaborates the information related to the human activities and object locations in the robot workspace. The overall scene is measured through motion capture sensors, the objects to be manipulated are detected using an RGB-D camera, while the data related to the operator action comes from the inertial sensor of a smart watch.

A teleoperated mobile manipulator proposed in [69] is controlled according to the posture for the operator’s hand. The tracking of the operator’s hand is achieved by employing a Leap Motion sensor, in which a Kalman filter is used for the position estimation while the orientation is computed by a particle filter. A similar contactless hand gesture recognition system is presented in [70] for safe HRI. This multi-modal sensor interface uses proximity and gesture sensors, and it can identify real-time hand gestures to control the robot platform. An ANN is used for the recognition of hand gestures.

Other approaches, such as the one presented in [71], can work along with a human through an admittance interface, allowing conjoined action. If the human is not in close proximity, the mobile manipulator can perform its routine work autonomously. In particular, the admittance interface is a mechanical connection from the robot hand to the human wrist and transmit the interaction forces of the human to the robot to perform conjoined movements. When the human needs assistance, it is possible to “call” the robot using the armband that recognizes the gestures of the human operator.

### 2.4. A Brief Overview of HRP in Industry

Presently, most of the sensors used for robotic systems to perceive the environment and the human operators are of vision type. In particular, in the field of human collaboration with manipulators, the most used sensor is the RGB-D camera. Indeed, the use of new types of sensors may require huge effort to define new algorithms and exploit their characteristics. Moreover, the already developed obstacle detection algorithms would need to be rethought to work with different data types. An interesting new vision sensor is proposed in [72], as Dynamic and Active-pixel VIsion Sensor (DAVIS). This novel sensor seems to have great potential for high-speed robotics and computer vision applications and incorporates a conventional global-shutter camera with an event-based sensors in the same pixel array, allowing the combination of their benefits as well: low latency, high temporal resolution, and very high dynamic range. However, for the moment more algorithms should be required to fully exploit the sensor characteristics and cope with its unconventional output, which consists of a stream of asynchronous brightness changes (called “events”) and synchronous grayscale frames. In those applications in which the employment of vision sensors is not sufficient or accurate, other kinds of sensors are used instead.

For what concerns IMRs, applications involving human perception mainly exploit laser range finders, to perceive the environment (humans included), usually combined with a vision sensor to perform data fusion. The massive use of laser range finders for human-perception goals is expected and justified, as it is a sensor typically present on IMRs both for obvious navigation requirements and for industrial safety guidelines (safety-rated scanners).

In the same way, mobile manipulators exploit predominantly laser sensors for navigation, while vision sensors are mainly used for the manipulator to perceive the human operator, in particular, to have some visual guidance and be able to imitate the movements of the human. Most of the vision sensors used in mobile manipulators are of RGB-D type, since they give accurate information related to the image and depth of the detected object. An alternative way for the robot to perceive the human actions is based on the use of an inertial sensor, attached to the human wrist to detect motions, and let the robot to predict and react according to the human movements.

**Figure 2:**
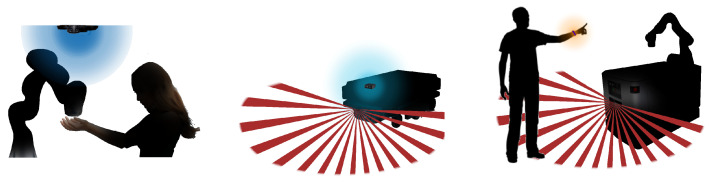
Relevant sensors for HRP within the industrial context. Vision sensors are highlighted in blue, safety laser scanners rays in red, and wearable sensors in orange.

Figure 2 gives a visual overview of the most relevant sensors used for HRP depending on the robot type, according to the authors’ research.

**Figure 3:**
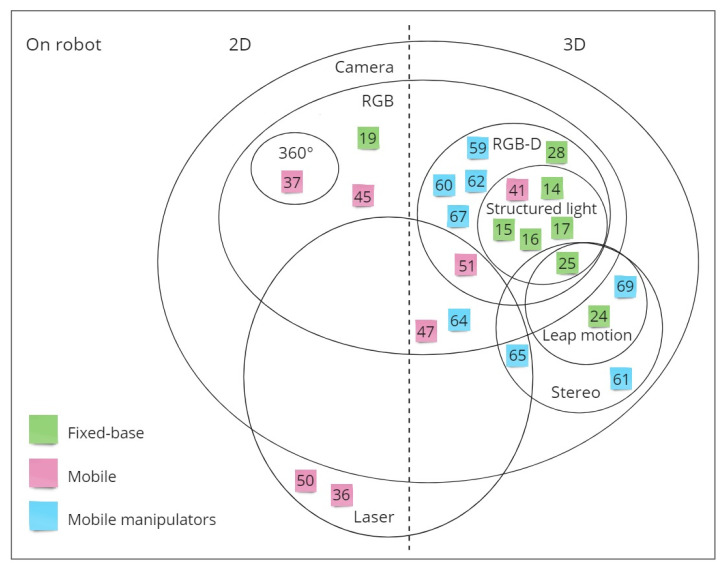
Most relevant sensors for HRP which can be found on the robot.

**Figure 4:**
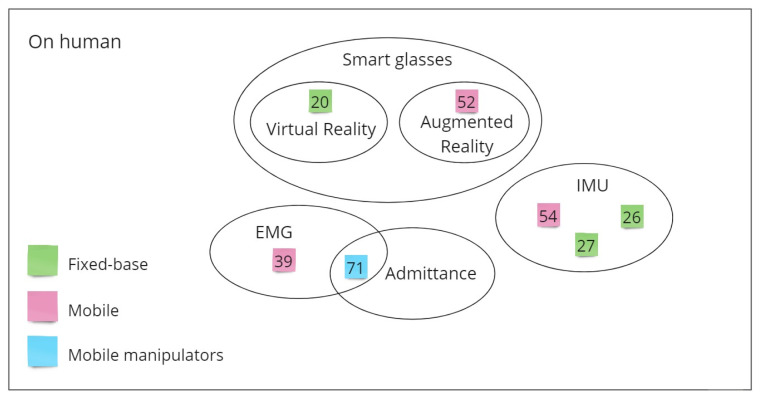
Most relevant sensors for HRP which can be found on the human operator.

To provide a summarizing overview of the relevant sensors and methodologies obtained from the described state of the art analysis, the following material is introduced:

- Figure 3 and Figure 4 aggregate the sources based on the employed sensors types, also carrying information about the used robot type. It is worth noting that by splitting sensors according to their presence on robots or on human operators, it is clear how (based on what has been analyzed) the sensory equipment for HRP are currently mainly positioned on the robot counterpart.
- Table 1 aims to enrich the overview presentation and ease the reader consultation, focusing on algorithms to implement HRP.

Although it is true that the provided material is a useful tool for getting a taste of the trending sensors and algorithms in HRP, it should be considered that it is limited to the authors’ research and, for this reason, it may not be exhaustive.

In the light of the authors’ analysis of sensors and algorithms combinations for HRP in the industrial context, human–robot perception seems to be inevitably linked to robot-human perception: each information on the human partner behavior is perceived by a robot, interpreted and transformed into action but, at the same time, human reaction to the presence of a robot is affected by the perception the operator has of the robot itself. An optimal reciprocal perception is however not easy to implement, given the lack of a common ground for cognitive skills among humans and robots, which affects the interaction. The robot not only has to detect the human presence but also to understand the context of collaboration, with the aim of effectively assisting human collaborators to improve productivity of the overall collaborative system while maintaining safety.

To achieve a comparable level of cognition among humans and robots, multi-modal sensor fusion is the preferred solution for the robot perception, either when considering environment perception and human perception, which are obviously interlinked. Data fusion, is a key module for autonomous systems to implement perception. Multi-modal data is analyzed at a raw level for fusion processing and then interpreted at a higher level to identify relevant features. Through multi-modal sensor fusion, the sensor performance can be enhanced exploiting data fusion. The latter can potentially bring out interesting information which, if single-source data only were considered, would have not emerged. This allows the implementation of a more informed perception of the environment and the humans within it.

Furthermore, what emerged is that safety is one of the factors leading the choice of algorithms and sensors: their combination must aim at satisfying safety conditions suggested by standards. Moreover, along with safety, also an appropriate interface plays a significant role when developing HRC tasks.

It is clear that where sensor accuracy is lacking, algorithmic complexity aims for compensation, to achieve an overall reliable interaction. As can be easily inferred, the balance among accuracy of the sensor and algorithm computational effort strongly depends on the available resources and on the application requirements.

## 3. Proofs of Concept for Future Applications of Perception Technologies

### 3.1. Collaborative Fixed-Base Manipulator: A Proof of Concept (POC)

Here a solution is proposed to enhance the HRC in applications involving fixed-base collaborative robots. This solution aims at paving the way for overcoming the current limits related to sensor accuracy and fast execution of the obstacle detection algorithms. We address the issue of human safety by using an RGB-D sensor in a two-fold way within a collision avoidance strategy. The problem of human and obstacle detection for collision avoidance is handled defining two different working ranges, depending on the operator proximity to the robot:

- Within the robot workspace, human safety is ensured by a collision avoidance strategy based on depth sensor information.
- Outside the depth sensor range, the human presence is detected thanks to a different processing of the RGB image only, performed by a YOLO CNN.

**Figure 5:**
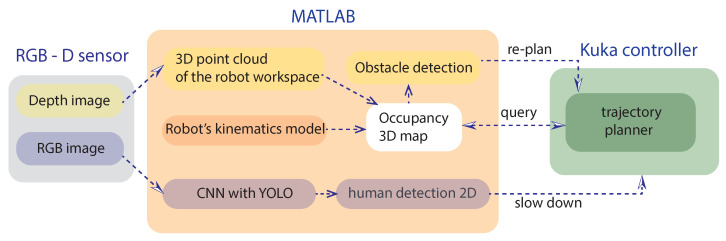
Architecture setup for obstacle detection at different working range. Data stream, processing and communication between sensor node and robot controller are showed.

**Figure 6:**
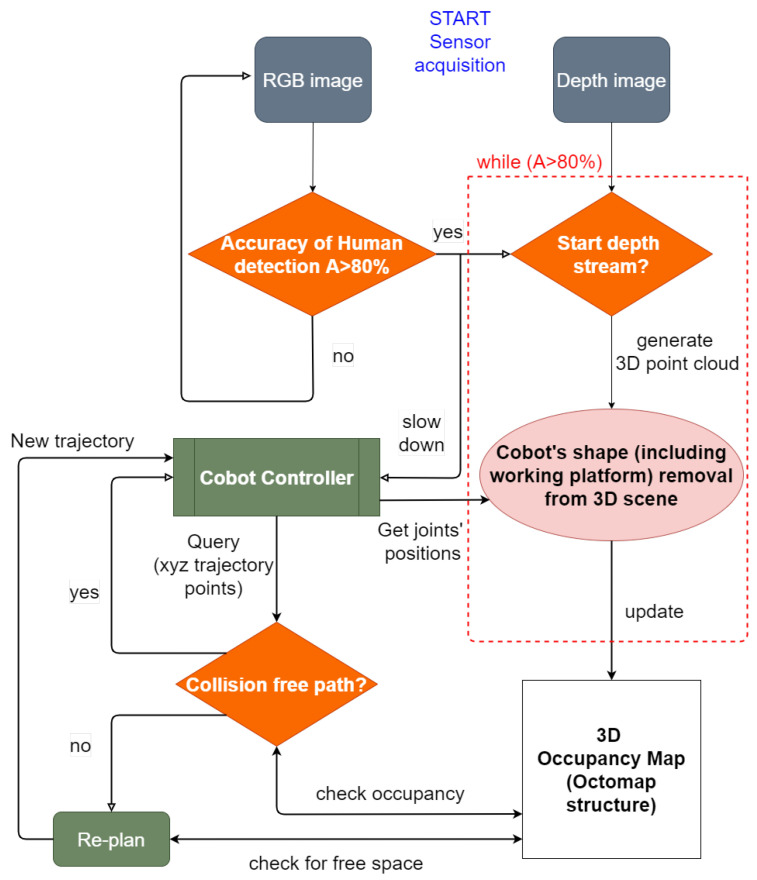
Block diagram representing the logical workflow and algorithms’ interaction of the POC.

CNNs were selected given their well-known good results when performing real-time human detection [73] and their capability to understand rich and complex features without the need to design features manually [74]. In this way, when humans are far from the robot working range, a-pre-safety condition can be enabled without affecting the robot tasks, until the human comes in proximity of the robot’s outer working range, and the robot starts moving slowly, because it is aware of a nearby human presence. The robot is not aware of the human’s exact location in the 3D space until he/she reaches the depth sensor range and his/her position can be traced. The YOLO CNN can detect the human presence even at distances greater than 10 m, using the RGB-D sensor with a good accuracy (A≈ 70–80%), as reported in [38]. For this reason, an outermost range is not exactly defined: the possibility to detect humans in long-range depends only on the sensor Field of View (FOV). At the same time, it is not necessary to slow down the robot if a human operator is walking 10 m away from the robot. Therefore, the RGB-D sensor is positioned to monitor a reasonable outer working range, and a threshold on the accuracy is defined to slow down the robot only if necessary. The space to be monitored is set as a trade-off between the need to guarantee the operator safety and the amount of free space to be left around the robot, depending on the specific application. The combination of these two methods and their related algorithms allows a suitable pathfinding and obstacle avoidance in the robot working space, taking into account also the human presence in the whole scene. The proposed approach will ensure the safety of the human in the robot workspace by combining obstacle detection algorithm with the force limitation policies already implemented on collaborative robots (such as the ones included in the KUKA iiwa robot). The overall high-level architecture is presented in Figure 5. In addition, the logical loops of the proposed architecture are presented in Figure 6. The decision flowchart better shows the logical interaction between the object detection and collision avoidance algorithms proposed for this POC.

More details about the application are given hereafter through a preliminary feasibility study, aiming at showing a wider usage of an industrial sensor generally employed for robot grasping applications. In particular, the potential of the Revopoint 3D Acusense RGB-D camera has been evaluated by analyzing the pros and cons in a possible HRI application. The selection of the RGB-D camera has been based on the suggestions reported in [18], where different types of RGB-D cameras are compared, and useful information regarding the choice among the most used in the research field can be found. Basically, the RGB-D camera has been chosen mainly comparing the following features: resolution, accuracy, sensor working range, frame rate and FOV. This kind of sensor has a depth working range from 20 mm to 2 m, with an accuracy of ±1 mm on measurements taken from 1 m to 2 m. Further details regarding the technical specifications can be found in Table 2. A fixed tripod mechanism supports the RGB-D camera to detect the presence of obstacles or humans in the robot workspace. The sensor provides RGB and depth space aligned images. These measurements are useful to generate both 3D point cloud of the working space and RGB images to train the CNN.

**Figure 7:**
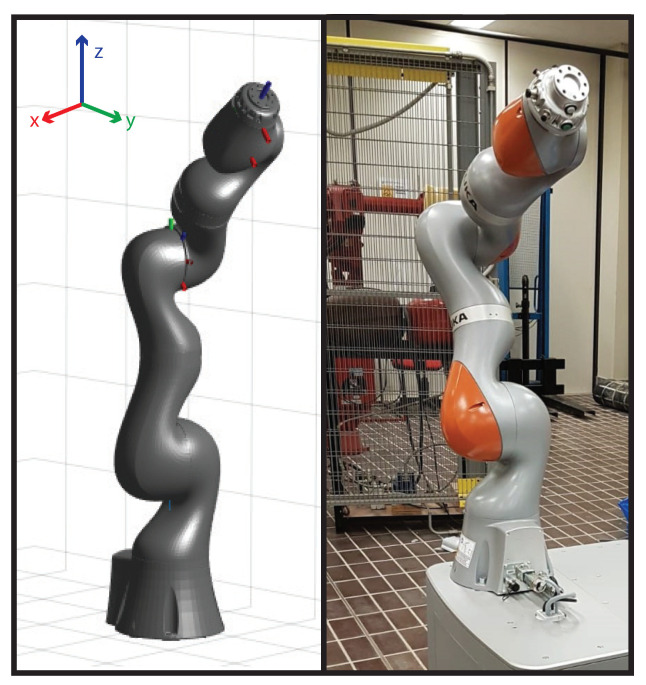
Simulated (**left**) and real (**right**) KUKA collaborative robot used on the proposed application.

**Figure 8:**
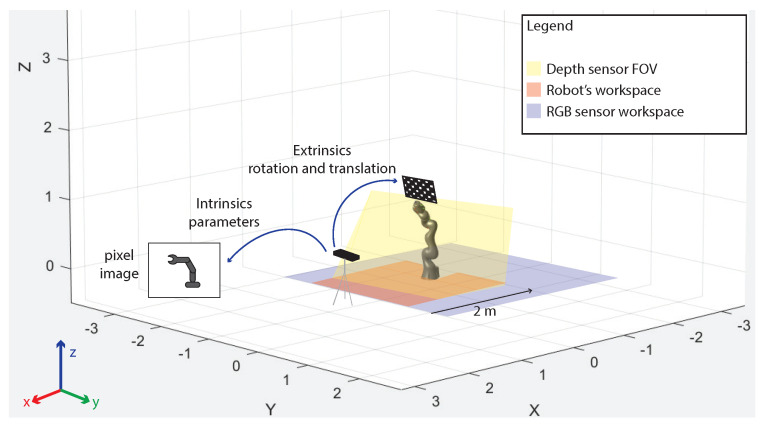
Schematic view of the calibration process with representation of sensor workspace and robot workspace. The axes measurement units are assumed in m.

**Figure 9:**
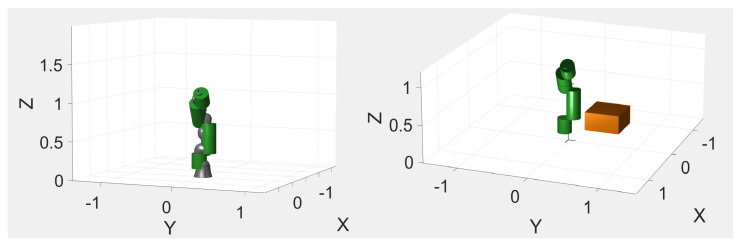
(**Left**) Mesh primitives (green colored) representing bounding volumes of collision envelope overlapped to the robot model. (**Right**) Collision envelope meshes attached to the robot structure using DH convention and collision box. The axes measurement units are assumed in m.

The proposed setup consists of the above mentioned RGB-D camera installed in proximity of the outer working range of an industrial collaborative robot (KUKA iiwa), which has been modelled to perform some preliminary tests in simulation, as shown in Figure 7. Through the kinematic model of the robot, developed according to the Denavit–Hartenberg (DH) convention, and through the instantaneous readings of the joint positions provided by the robot controller itself, it is possible to instantaneously know the occupancy space of the robot in the 3D environment. The projection of the 3D occupancy space in the pixel coordinates of the RGB-D sensor is achieved through the camera calibration process (Figure 8). This allows the identification of the robot shape in the RGB image, which is then removed to avoid that the robot itself is considered to be an obstacle in the occupancy map. The kinematics model of the robot is defined as a tree structure, and bounding volumes are attached to the robot links. More in detail, cylinder meshes are considered to be bounding volumes of collision envelopes and attached to the robot structure, as shown in Figure 9. Once acquired the joints’ positions, using the DH convention it is possible to define the position of the robot bounding volumes in the 3D workspace. By using the camera’s extrinsic parameters acquired during the calibration process of the RGB-D sensor, the robot position in the 3D space can be projected onto the image pixel coordinates. Then, the pixels belonging to the robot and to the static objects of the scene are simply removed. In such a way, the 3D map is updated only when new dynamic obstacles are detected by the CNN and by the depth sensor in the inner workspace.

The novelty of the proposed approach relies on:

- The use of a high accuracy sensor, commonly mounted on the robot arm to identify the shape of the object to grasp.
- The data processing process, which uses the RGB and depth information separately.

As previously mentioned, the RGB image is used for long-range human detection, while the depth image is used to accurately acquire 3D point cloud data in the short-range (see the RGB and the robot workspace in Figure 8). The 3D point cloud data are registered using the OctoMap structure to achieve a faster execution of the collision avoidance algorithms [75].

**Figure 10:**
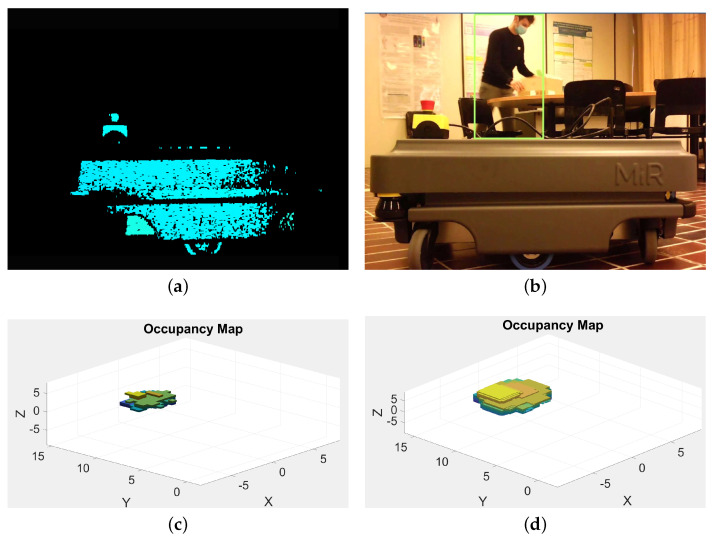
RGB-D data processing and occupancy map reconstruction. (**a**) Depth space image acquisition of Mir200 AGV. (**b**) Human detection in long-range with YOLO CNN based on RGB image. (**c**) Occupancy map created with Octotree structure, the axes measurement units are assumed as 1 unit = $10^{-1}$ m. (**d**) Occupancy map with inflation radius of 0.2 m, the axes measurement units are assumed as 1 unit = $10^{-1}$ m.

Concerning the comparison with other low-cost RGB-D sensors, the Acusense RGB-D camera has very good accuracy in a short-range (2 m), allowing the obtaining of richly detailed scanned images, with the only shortcoming of being heavy, from a computational point of view. Given that the depth sensor working range is sufficient to monitor the robot workspace, acquisitions made within it result as reliable. However, obstacles at a greater distance cannot be detected in a reliable way. To overcome this limit, the RGB image is processed in parallel by a CNN, which makes use of the YOLO structure to detect humans beyond the working range of the sensor, while the depth image is used to check the robot workspace. The inner workspace of the robot is related to its working platform. The platform is considered to be a static object of the scene, and the relative information is removed from the 2D depth image. In this way, during the robot motion, a new query is performed only if the 3D occupancy map is updated due to the detection of dynamic obstacles. The process to remove the robot and the static objects from the 2D depth image unavoidably influences the computational time; however, using this method, the updates of the inner workspace can be performed about every 200 ms with an a-priori selected resolution of the 3D occupancy map. On the contrary, keeping the static objects in the inner workspace, a continuous query process should be performed. The computational time required by the query process depends on the chosen 3D map resolution: the deeper the 3D map, the more the query process affects the computational time. In Figure 10a a depth image acquisition is shown, while Figure 10b shows the application of the long-range human detection on the RGB image. This example has been developed in a laboratory setup, considering a moving Mir200 AGV as an obstacle. During the short-range processing, the depth images of the robot surrounding environment are modelled using an occupancy 3D map based on the Octree structure (Figure 10c). This method allows for fast path planning and obstacle avoidance, thanks to a more lightweight information with respect to classic 3D point cloud data. The Octree indexes the three-dimensional space, so that the occupancy state of each region can be determined. In such a way it is possible to continuously monitor the distance between the robot and the occupied regions (obstacles). An updating rate of 500 ms has been achieved for the 3D map, by using an AMD Ryzen 5 (4000 series) processor with 8 GB RAM and integrated GPU. Moreover, before engaging any trajectory, it is possible to check if some obstacles are already present along the robot path, by sending a query to the Octree structure, which comes in handy during the path planning phase. A further feature of the Octree concerns its multi-resolution representation capability. Every 3D point cloud is registered in the Octo-structure and, by limiting the depth of a query, multiple resolutions of the same map can be obtained at any time. This process allows choosing the needed resolution, also allowing the collision avoidance algorithms to run as fast as possible. In our case, a preliminary analysis identified 10 cells/m${}^{3}$ as the best 3D map resolution. Indeed, a higher resolution, e.g., 100 cells/m${}^{3}$, would exponentially increase the execution time of the algorithm. A single, random query on a tree data structure containing *n* nodes with a tree depth *d* can be performed with a complexity of O(d)=O(logn), as reported in [75]. Therefore, much more computing power is required to achieve the same performance in terms of the algorithm execution time. Using a dedicated GPU to elaborate RGB and 3D point cloud information, can be a valid solution to deal with such data processing issues. However, in this POC no dedicated GPU has been used to process the data retrieved by the RGB-D sensor. Using this approach, there is no need to define bounding volumes for the obstacles to check their distance from the robot. Indeed, it is sufficient to define an inflation radius around any occupied region (Figure 10d). In such a way, defining a safe inflation radius (for instance, 0.2 m in our case), it becomes easy to keep a safe distance between the robot and the obstacles. When the robot position coordinates and the additional safety radius overlaps an occupied location of the 3D map, it means that the robot is too close to the object and a new path should be re-planned. As a first approach to obstacle detection within a short-range distance, no distinctions were made between generic obstacles and humans. Indeed, the depth space information is simply used to check free space in the environment.

#### Potentialities, Preliminary Validation and Feasibility of the POC

Some considerations can be made from the preliminary results already achieved for the proposed POC.

Elaborating the RGB and the depth information separately allows the definition of two different priorities for the task and to parallelize the computation. The depth sensor loop starts when the CNN detects a human that can be in the inner or in the outer area of the workspace. When the human is inside the robot workspace, the 3D occupancy map is updated with new information. On the other hand, if the human is still far from the robot inner workspace, the depth sensor does not perceive new obstacles and the occupancy map does not change. It must be also underlined that if the robot must collaborate with AGVs or other autonomous systems, the CNN can be trained to classify not only humans but also other dynamic obstacles.

The execution time is evaluated as the time elapsed for the two loops, namely obstacles detection and collision avoidance. Using a different hardware setup, the two loops may be speeded up. The current execution time required by the entire code of the POC is about 500 ms, which represents a good performance in comparison with other collision avoidance algorithms. Such a value represents the maximum elapsed time for the entire process. For instance, in [19] the obstacles detection is performed in 2D, assuming a constant height and using two low-cost RGB cameras; the working cycle is set to 45 ms but without considering humans as obstacles. The depth information is the one requiring more computational time and in the afore cited work it is not used. Moreover, in [76] the scale-invariant features transform (SIFT) descriptor and Multi-scale Oriented Patches (MOPS) are combined to elaborate the 3D information of the obstacles. The edges and corners of the objects are then extracted using MOPS, then the 3D spatial information of the MOPS points is extracted. This approach is related to Unmanned Aerial Vehicle (UAV) obstacle detection and has a computational time of 577 ms. Lastly, in [77], an integrated solution is presented for real-time planning in changing environments using 3D sensor data. The path planner is based on Dynamic Roadmaps and is implemented on the mobile robot platform Care-O-bot 3. The authors state that real-time results were critical to be achieved for the specific implementation. They propose a heuristic optimal search, enabling a fast connection of the start and goal configurations to the roadmap. The experiments show that the integrated solution can calculate a collision-free path for the 7-DOF manipulator of Care-O-bot 3 within 100 milliseconds, but nothing is said about the time required for sensor acquisition and processing of the 3D data, which is in general the most time-consuming. Unfortunately, very few works present results regarding the execution times required by the collision avoidance and object detection algorithms.

The POC of the proposed technique, comprising the implementation of the robot model, the sensor acquisition, the actions computation, the obstacle detection and mapping, has been developed in the MATLAB environment, establishing communication with the KUKA controller by means of TCP/IP connection [78]. The feasibility of the methodology and the robot interaction were preliminary tested in simulation, for safety reasons. The RGB-D sensor algorithms for obstacles detection were implemented, running in parallel with simulations. The information retrieved by the sensor is imported into the virtual 3D scene where the cobot is modelled. The collision avoidance strategy is then validated in simulation by testing the interaction of the developed codes. This is possible by using mesh primitives (e.g., cylinders) as bounding volumes of collision envelopes to model the robot and the objects in the scene, as shown in Figure 9. The simulation automatically stops if a collision occurs. This preliminary study shows interesting results but, to achieve a complete robot awareness of the whole 3D workspace, a multi-sensor architecture with at least three RGB-D sensors is suggested. In fact, blind spots behind the obstacles and shadows can disturb the reading of a single sensor. In addition, a proper amount of environmental light is very important for a correct depth sensor acquisition, but this should not represent a serious limitation for the proposed approach in practice, since it is required only in a limited area, corresponding to the robot workspace. A brief recap of the strengths and weaknesses of the proposed POC are summarized below:

1. pros:
  - Possibility of defining two monitoring areas (long and short-range) and therefore greater safety for the human operator.
  - Possibility of processing RGB and depth images separately to structure a flexible monitoring technique for dynamic obstacle avoidance.
  - Data processing methodology that reduces the amount of information, allowing efficient real-time functioning.
  - No need to calculate the distance between objects using an inflation radius to ensure a safe distance between obstacles and robots.
2. cons:
  - The depth sensor is sensitive to reflective surfaces.
  - Depth sensor measurements are reliable in a limited range sufficient to monitor the robot workspace only.
  - The RGB-D sensor is not suitable for installation in dusty industrial environments or with high variability of brightness.
  - Need to have more than one sensor to cover any blind spots and to monitor the 3D robot workspace.

In future developments, the Gazebo environment will be used to validate particular use cases and to test the algorithms in a more realistic scenario. The last step of validation will consist of implementing the entire approach by merging sensor information and cobot controller reading using a dedicated computing unit. Furthermore, two additional RGB-D sensors will be included in the experimental setup, to avoid blind spots in the robot workspace.

### 3.2. Collaborative Sen3Bot: A Proof of Concept

This section describes a POC of a collaborative behavior implementable upon the Sen3Bot mobile agent, the main element of the Sen3Bot Net [29,79]. The Sen3Bot is an IMR (an AMR, specifically) enabled the pursuit of an assigned task within a space shared with human operators. Beyond the standard tasks of AMRs, the Sen3Bots are given the main role of serving as *meta-sensors*: they themselves represent a distributed network of sensors supporting a fleet of traditional industrial AGVs, informing it about the human presence in areas at risk of hazardous situations. In fact, the Sen3Bot has human detection and avoidance capabilities, tested on a real demonstrator [80], allowing for cooperation. First, to lay the groundwork for a more collaborative approach, the Sen3Bot behavior could be improved by incorporating in the human avoidance algorithm relevant factors coming from the Proxemics Theory, e.g., speed adjustment and direction of approach.

It is undeniable that safety is the main design requirement for IMR sharing the workspace with human workers [81]. Please note that for the proposed POC, the working area subdivision according to a critical level will be considered (as described for the Sen3Bot Net). The definition of such areas was mainly inspired by ANSI safety standard guidelines for driverless vehicles [82], whose corresponding standard in Europe is the ISO 3691-4:2020 “Industrial trucks—Safety requirements and verification—Part 4: Driverless industrial trucks and their systems”, which specifies safety requirements and verification means for driverless industrial trucks, including AGVs and AMRs [58].

Nevertheless, what emerged is that safety standards struggle to keep pace with the fast evolution of collaborative/cooperative AMRs. Current guidelines limit the flexibility that would be potentially achievable with new AMRs. For instance, the fact that AMRs paths do not need to be pre-defined allows the removal of the limitations given by the physical installation constraints imposed by many traditional AGVs; however, standards suggest that AMRs paths should be marked, which hinders a fast reconfiguration.

To overcome this limitation, an online supervisory planning algorithm for mobile robots was presented in [83]. Given a static map, the mobile agent can follow a virtual safe path in an industrial-like scenario and the trajectory is re-planned when a human operator is in close proximity to the robot in motion.

Even though in this cooperative scenario the human operator is safe during the robot motion, it is worth observing that the inflation radius assigned to the identified human can possibly be so large that the robot may have problems to move in narrow spaces. An improvement to this system could be to identify human operators to distinguish trained operators from the general staff and, in the first case, reduce the safety radius surrounding the operator. Further details will be explained hereafter, illustrating a general idea to improve the system and provide collaborative capabilities to a Sen3Bot through the implementation of safe interactions, exploiting and improving HRP capabilities intended as the interpretation of data at a behavioral level.

To illustrate the idea, the following assumptions will be considered:

1. In the light of the envisioned collaborative extension:
  - If the area has the highest criticality level (area of type 1), the AMR must work in cooperative mode, implying that conservative avoidance of humans is implemented.
  - If the area has the medium criticality (area of type 2), the AMR can switch between two modes, cooperation or collaboration with human operators.
2. The working space taken into account is an area with criticality level equal to 2, i.e., a sub-critical area, corresponding to a zone that includes cobots workstations and manual stations where human operators are likely to be present, but expected to be mostly static.
3. In such a sub-critical area, the human operators are assumed to be mainly trained ones, i.e., they are aware that the area is shared with AMRs and know how to interact with them. Operators of this type are identified by a tag, e.g., a QR code, on the front and the back of a wearable leg band.
4. According to the Sen3bot Net rules, if a critical area of type 2 is foreseen to be crossed by an AGV, two Sen3Bots are sent to the scene if the human operator is moving within the environment.

Collaborative extensions of the Sen3Bot Net can then be developed in the scenario described hereafter, under the above listed assumptions. Two Sen3Bots monitor the scene in a sub-critical area: such a redundancy ensures the operator safety by taking into account its dynamic behavior, understanding if he/she needs some assistance from the mobile agents. In principle, both AMRs can act as collaborative mobile robots. Nevertheless, once on the scene, only one enters a *wait4col* state, i.e., an idle state where the AMR waits for a triggering command from the operator and signals its state through a visual indicator, e.g., a led signaling the current AMR active mode. As the operator is recognized as trained, proximity rules can be less conservative: the robot can reduce the inflation radius around the detected human, since the latter is supposed to be aware of the former’s behavior.

If the operator needs assistance from one of the Sen3Bots, he/she will have to perform a pre-defined sequence of movements:

- The operator approaches the AMR in *wait4col* idle mode.
- The operator stops at a fixed distance *Df* in front of the mobile robot letting it read for a given time *Tf* the front leg band tag, which contains relevant information (such as the operator ID).
- If the operator turns around, letting the robot read the back tag for a time *Tb*, then the collaborative mode of the Sen3Bot is activated.

**Figure 11:**
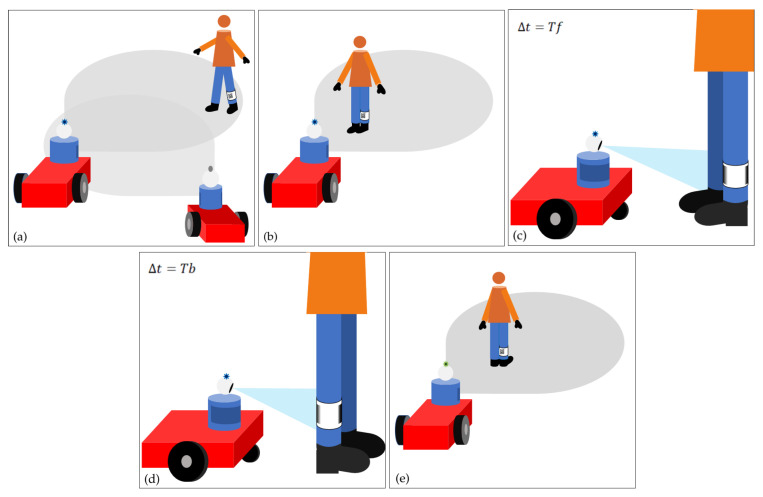
Workflow for activating the collaborative mode of the Sen3bot. The grey area represents the intersection of the vision and laser sensors FOV. (**a**) A human operator within the monitored area of type 2 needs assistance from one of the Sen3Bots. (**b**) Given the led blue color, the operator identifies the Sen3Bot ready for collaboration. (**c**) The human operator stops at a distance *Df* allowing the robot to scan its front QR code. (**d**) The human operator turns around allowing the robot to scan its back QR code. (**e**) The green indicator light indicates that the mobile robot entered the collaborative mode.

Feasible values for *Df* are in the range of 0.5 m–1 m, while *Tf* and *Tb* can be chosen between 2 and 3 s. Please note that the above sequence can trigger several collaborative applications, e.g., Follow-Me, assistance with materials or tools. Furthermore, when the operator executes such sequence for the second time in front of the same robot, the collaborative mode of the mobile agent is deactivated. In this case, the Sen3Bot can be re-assigned a new task, for example monitoring a different area. Figure 11 shows the described behavior.

**Figure 12:**
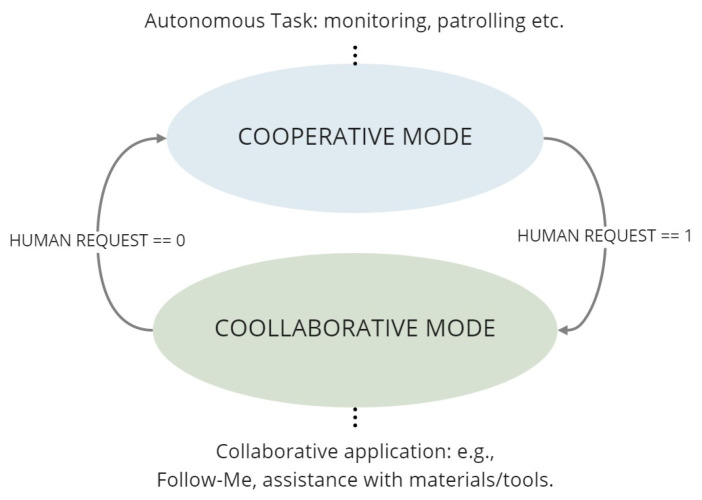
Modes switching schema for a Sen3Bot monitoring an area of type 2, enabled to wait for collaborative task triggering, i.e., with *wait4col* ==1.

It is worth noting that the Front-Back sequence intends to emulate the human interaction that is likely to take place when two persons are conversing. In this way, the mobile agent can understand this common human intention demonstration, allowing a narrowing of the gap between the different cognitive skills between human and robots. Please note that the time and distance parameters considered in the sequence take into account a suitable tolerance. A schematic representation of the Sen3Bot modes characterizing the proposed collaborative approach is given in Figure 12. In particular, the HUMAN REQUEST flag is set by default to 0, since the robot starts with the cooperative mode and becomes 1 when the conditions of the first Front-Back sequence are valid, allowing the robot to switch to the collaborative mode. The flag is reset to 0 when the human operator performs a valid Front-Back sequence for the second time to the same robot.

The proposed idea could speed up the time required for human operators to be trained to use mobile agents within the industrial workspace, since the interaction with them recalls a human-like behavior. Even a non-robotic expert could employ the mobile agents, enabled by a user experience similar to the manual guidance of cobots, which streamlines the robot programming and therefore the production line setup. The idea of wearing a leg band with a tag represents a low-cost solution for identifying operators, to track their activities and thus ensure safety within the industrial environment. In fact, a tag such as a QR code may contain all the needed information for the robot and the overall system. However, it is not possible to state how robust the solution is, at least, not until a real implementation in a real industrial scenario is tested. Furthermore, running an algorithm that identifies the human operator, tracks his/her location within the map and at the same time filters and processes relevant information coming from the QR code, may need a high-performance system for computing the perception algorithm.

Finally, taking into consideration the current implementation of the Sen3Bot agent, such envisioned collaborative module could be implemented by taking advantage of tools for tag recognition such as Zbar, for which a ROS (Robot Operating System) wrapper node is available [84]. Also, other tools are present as Open-Source material, mainly based on the OpenCV library, whose ROS compatibility is well-established as well. Please note that this additional collaboration module would not imply the need for further sensors, since the already available IP camera video stream would allow the application of the afore-mentioned vision tools.

## 4. Conclusions and Future Trends

Human intention recognition is a trending topic within the HRI research field, especially for industrial environments. Human–robot perception and the prediction of possible humans’ unsafe conditions will be a fundamental enabler for anticipating human operators behavior and needs, to implement proactive collaboration among humans and robots. In advanced manufacturing plants, many applications require HRC operations, where humans and robots perform joint tasks or share the same environment. These robots should then be able to adapt their motion to the human presence and, if required, accomplish cooperative tasks. From the other side, the shared workspace between robots and humans may decrease productivity, if the robot is not aware of the human position and intention. Future smart production processes will have to cope with the need to guarantee the satisfying of production KPIs (Key Performance Indicators), and the new robotic systems integrated in such processes will have to be compliant with these requirements, too [85]; therefore, not only boosting HRP will help to satisfy safety requirements, but it will also contribute to hit ideal overall KPIs. An efficient collaboration with humans will be a fundamental element for various production processes, which are still only partially automated. The manual execution of some operations within the manufacturing process, as well as the presence itself of human operators in the environment shared with robots, will have to become an added value for the quality of the results, and not a potential cause of low efficiency. This goal can be achieved only through a proper choice of sensors and techniques, suitable for each particular kind of robotic system and application. This work has surveyed the main sensors and techniques, currently available to perceive and react to the presence of human operators in industrial environments, with reference to the various types of robotic systems commonly used in industry. On the basis of the carried-out analysis, some general considerations can be drawn:

- Vision sensors are fundamental to handle the human presence for any kind of robotic system, and in particular the most used one is the RGB-D camera.
- The combination of different kinds of sensors, possibly located on the robot and/or the human operator, can allow new types of collaboration and applications.
- Laser sensors are often used for human-perception purposes in combination with the vision ones in the case of mobile agents and manipulators, since they are typically present and used for navigation.
- The use of new, non-standard sensors is still limited, mainly due to the critical management of their somehow unconventional outputs.
- Most of the methods involved in HRP are enabled by the recognition of objects or human behavior, especially taking advantage of artificial intelligence algorithms.
- New HRC applications can be envisaged also with sensors more commonly available, thanks to an innovative use of the information provided by them, as in the first presented POC, or through a coded collaborative HR behavior, as in the second POC.

It must be finally underlined that several even smarter industrial HRC applications can be envisioned, provided that an efficient multi-modal sensor fusion can be guaranteed, possibly also including those sensors and methods that are currently mostly adopted in other contexts, such as assistive service robotics, agriculture and robotic surgery.

## Figures and Tables

**Table 1:** Most relevant algorithms for HRP applications, grouped according to how the perception is implemented.

**Collision Avoidance**	
[12]	Collision prediction using time-invariant models and neural networks on signal processing.
[14]	Collision prediction based on over-segmentation using forward kinematic model.
[15]	Collision avoidance through generation of repulsive vectors.
[16]	Collision avoidance and re-planning algorithms.
[17]	Collision avoidance exploiting skeletal tracking and positioning of the user.
[19]	Collision avoidance using color detection and allows online path planning.
[20]	Collision avoidance through virtual forces applied on the manipulator.
[22]	The algorithm imposes velocity limitations only when the motion is in proximity of obstacles.
**Aware Navigation**	
[36]	The robot travels in virtual areas defined a-priori by users.
[40]	Social momentum, teleoperation and optimal reciprocal collision avoidance are used as navigation strategies.
[45]	The planning model is based on RNNs and image quality assessment, to improve mobile robot motion in the context of crowds.
[49]	An autonomously sensed interaction context that can compute and execute human-friendly trajectories.
[50]	Robot navigation takes into consideration the theory of proxemics to assign values to a cost map.
[51]	A confidence index is assigned to each detected human obstacle, enclosed in a 3D box, to avoid it accordingly.
**Environment Representation**	
[41]	The algorithm adapts to continuous short-term and long-term environment changes with focus on human detection, through feature extraction.
[42]	Scene matching through a representation learning approach that learns a scalable long-term representation model.
[62]	Object localization and tags recognition allow the robot to gather semantic information about the environment.
**Recognition of Objects and Behavior**	
[47]	The robot assistance is improved using adaptive sensory fusion.
[24]	Teleoperation using coded gestures recognition as input commands.
[25]	Simultaneous perception of the working area and operator’s hands.
[53]	Gesture control and eye tracking technologies are used by the robot to interpret human intentions.
[54]	The human motion here is registered and used for skill transfer purposes.
[61]	The motion of the collaborating human operator is monitored to enable specific robot actions
[63]	Gesture recognition is performed considering a convolutional representation from deep learning and a contour-based hand feature.
[64]	Human tracking is implemented and trained according to human body patterns.
[65]	Human detection and behavior recognition is implemented exploiting redundancy of sources to reconstruct the environment.
[67]	The information related to the human activities and object locations in the robot workspace are used for the approach.
[69]	The operator’s hand pose is estimated using a Kalman filter and a particle filter.
[70]	Real-time hand gesture recognition is implemented using a ANN.
[26]	The gestures used to command the robot are processed and classified by an ANN.
[27]	The motion of the human operator’s upper body is tracked, with a focus on objects manipulation.
[28]	Sensor data fusion algorithm for prediction and estimation of the human occupancy within the robot working area.
**Conjoined Action**	
[37]	The 360-degree scene is enriched by interactive elements to improve the teleoperated navigation.
[39]	Teleoperated navigation is implemented through a hybrid shared control scheme.
[52]	Enhanced perception-based interactions using AR for collaborative operations.
[71]	Interaction forces of the human are transmitted from the admittance interface to the robot to perform conjoined movements.

**Table 2:** Acusense RGB-D technical specifications.

Technology	Dual Camera Infrared Structured Light
RGB resolution and frame rate	2560 × 1600 @15 fps (8 M pixels)
	1600 × 1200 @15 fps (2 M pixels)
Depth resolution and frame rate	1280 × 800 @2 fps
	640 × 400 @10 fps
RGB Sensor FOV	H51° × V32° (8 M pixels)
	H42° × V32° (2 M pixels)
Depth Sensor FOV	H49° × V29°
Laser safety Class 1	820–860 nm
Operating environment	Indoor only
Trigger	External trig in/out

## Data Availability

No new data were created or analyzed in this study. Data sharing is not applicable to this article.

## Abbreviations

The following abbreviations are used in this manuscript:

CPPS	Cyber Physical Production System
CPS	Cyber Physical System
CPHS	Cyber Physical Human System
HR	Human-Robot
HRI	Human-Robot Interaction
HRP	Human-Robot Perception
FoF	Factory of the Future
HRC	Human-Robot Collaboration
RGB	Red Green Blue
RGB-D	Red Green Blue—Depth
LIDAR	Laser Imaging Detection and Ranging
3D	Tri-Dimensional
ISO	International Organization for Standardization
TS	Technical Specification
2D	Two-Dimensional
IMU	Inertial Measurement Unit
ANN	Artificial Neural Network
IMR	Industrial Mobile Robot
AMR	Autonomous Mobile Robot
AGV	Automated Guided Vehicle
YOLO	You Only Look Once
CNN	Convolution Neural Network
EMG	Electromyography
AR	Augmented Reality
RNN	Recurrent Neural Networks
OpenCV	Open Computer Vision
SAN	Social-Aware Navigation
MES	Manufacturing Execution System
EN	European Standards
2½D	Two-and-a-Half-Dimensional
RFID	Radio Frequency IDentification
ID	Identifier
FOV	Field Of View
POC	Proof of Concept
DH	Denavit–Hartenberg
GB	Gigabyte
RAM	Random-Access Memory
GPU	Graphics Processing Unit
SIFT	Scale-Invariant Features Transform
MOPS	Multi-scale Oriented Patches
UAV	Unmanned Aerial Vehicle
DOF	Degrees Of Freedom
TCP/IP	Transmission Control Protocol/Internet Protocol
ANSI	American National Standards Institute
QR	Quick Response
ROS	Robot Operating System
IP	Internet Protocol
KPI	Key Performance Indicators
